# Lipidomics reveals the potential mechanism of honokiol against adenine-induced chronic kidney disease

**DOI:** 10.3389/fphar.2022.1019629

**Published:** 2022-10-14

**Authors:** Xinhui Liu, Liwen Gao, Xi Huang, Ruyu Deng, Xian Wei, Jiandong Lu, Shunmin Li

**Affiliations:** ^1^ Department of Nephrology, Shenzhen Traditional Chinese Medicine Hospital, Guangzhou University of Chinese Medicine, Shenzhen, Guangdong, China; ^2^ The Fourth Clinical Medical College, Guangzhou University of Chinese Medicine, Shenzhen, Guangdong, China; ^3^ Shenzhen Traditional Chinese Medicine Hospital Affiliated to Nanjing University of Chinese Medicine, Shenzhen, Guangdong, China

**Keywords:** honokiol, chronic kidney disease, lipidomics, ultra high performance liquid chromatography, mass spectrometry

## Abstract

Honokiol (HKL), a biphenolic compound, is derived from the bark of *Magnolia officinalis*, which is used in traditional Chinese medicine for gastrointestinal complaints. HKL has diverse pharmacological activities and has protective effects in various disease models. However, the role and mechanism of HKL in treating chronic kidney disease (CKD) remain unclear. This study was designed to investigate whether HKL can alleviate CKD and the potential mechanism by which it acts. Male Sprague-Dawley rats were fed 0.75% *w/w* adenine feed for 3 weeks to induce CKD. HKL was administered by gavage at a dose of 5 mg/kg/day for 4 weeks. Using a special kit, serum creatinine (Scr) and blood urea nitrogen (BUN) were measured. To assess renal pathology, periodic acid-Schiff and Masson’s trichrome staining were conducted. Renal lipid profiles were analyzed by ultra-high-performance liquid chromatography/mass spectrometry (UHPLC/MS). The results showed that the administration of HKL reduced Scr and BUN and alleviated renal tubular atrophy and tubulointerstitial fibrosis in an adenine-induced CKD rat model. By using lipidomics, we identified 113 lipids (47 lipids in negative ion mode, 66 lipids in positive ion mode) that could be significantly reversed by HKL treatment in CKD rat kidneys. Most of these lipids belonged to the phosphatidylcholine (PC), ceramide (Cer), phosphatidylethanolamine (PE), and triacylglycerol (TAG) classes. Moreover, HKL improved fatty acid oxidation in the kidneys of CKD rats. In conclusion, this study found that HKL can protect against adenine-induced CKD, possibly through the regulation of lipid metabolism.

## Introduction

The global burden of chronic kidney disease (CKD) is growing and contributes to high morbidity and mortality (Kalantar-Zadeh et al., 2021). Dyslipidemia is a common clinical manifestation in CKD patients (Ferro et al., 2018). In turn, abnormal lipid metabolism associated with CKD contributes to the progression of CKD and the development of cardiovascular disease (CVD) (Noels et al., 2021), which is the leading cause of death in CKD patients (Thompson et al., 2015). Lipidomics provides an approach to systematically study lipids including their classification, abundance and biological functions. The use of lipidomics helps to find biomarkers for disease diagnosis and prognosis, and to monitor the response to drug treatment (Zhao et al., 2015). Thus, lipidomics is a powerful tool to study the pathogenesis of CKD and targets for drug intervention.

Honokiol (HKL, C_18_H_18_O_2_) is a biphenolic compound derived from the bark of *Magnolia officinalis*, that is used in traditional Chinese medicine for gastrointestinal complaints (Watanabe et al., 1983). HKL has diverse pharmacological activities including anticancer, antioxidative, anti-inflammatory, antidepressant, and neuroprotective effects (Shen et al., 2010; Talarek et al., 2017; Rauf et al., 2018; Wijesuriya and Lappas, 2018; Zhang et al., 2019). The renoprotective effects of HKL have been reported in sepsis-induced acute kidney injury (Li et al., 2014; Xia et al., 2019; Zhang and Xiang, 2019), renal ischemia/reperfusion injury (Yu et al., 2016; Park et al., 2020), lupus nephritis (Yang et al., 2020), and unilateral ureteral obstruction-induced renal fibrosis (Chiang et al., 2011; Quan et al., 2020). However, the role and mechanism of HKL in treating CKD remain to be determined. Previously, HKL was reported to attenuate lipid accumulation and lipotoxicity in hepatocytes to protect against non alcoholic fatty liver disease (Seo et al., 2015; Liu et al., 2021). In mice with cisplatin-induced acute kidney injury, HKL could improve fatty acid oxidation and renal function and reduce fatty acid deposition (Li et al., 2020). Moreover, HKL inhibited adipogenesis and promoted the browning of white adipose tissue that is induced by high-fat diets in mice (Ding et al., 2021). The above studies suggest that HKL regulates lipid metabolism. Since abnormal lipid metabolism is a hallmark of CKD, we hypothesized that HKL could correct lipid metabolism and slow CKD progression. To test this hypothesis, we established an adenine-induced CKD rat model and then treated the rats with or without HKL. Serum biomarkers of renal function, renal pathology, and lipidomics were utilized to evaluate the effect of HKL on CKD and the mechanism by which it occurs.

## Materials and methods

### Animals

The animal experiment was conducted according to the protocols approved by the Experimental Animal Ethics Committee of Guangzhou University of Chinese Medicine. A total of 18 8-week-old male Sprague-Dawley (SD) rats were purchased from Guangdong Medical Laboratory Animal Center (Foshan, China). After 1 week of adapted feeding, the rats were randomly divided into three groups of six rats each. Control rats were fed regular feed, while those in the CKD and CKD + HKL groups were fed 0.75% w/w adenine feed for 3 weeks. At the same time, rats in the CKD + HKL group were treated with HKL (5 mg/kg/day) by gavage for 4 weeks. All rats had free access to food and water. Blood and kidney samples were collected shortly after the experiment ended.

### Measurement of serum biochemical indices

Serum was obtained by centrifuging blood samples at 2000 rpm for 10 min after coagulation. Serum creatinine (Scr) and blood urea nitrogen (BUN) levels were detected with specific kits (StressMarq Biosciences, British Columbia, Canada) according to the instructions. Alanine transaminase (ALT) and aspartate transaminase (AST) detection kits were used to test serum ALT and AST levels (Jiancheng Bioengineering Institute, Nanjing, China).

### Histopathology

We fixed the kidney tissues with 4% paraformaldehyde overnight at 4°C, dehydrated them with an alcohol gradient, and embedded them in paraffin. Wax blocks containing kidney tissue were cut into 4 µm sections and stained with periodic acid–Schiff (PAS) and Masson’s trichrome. Representative pictures were captured by an Axio Imager M2 microscope and ZEN 2.6 software (Carl Zeiss, Jena, Germany). Renal tubular injury was scored based on tubular epithelial cells atrophy and shedding and tubular dilation. No tubular injury = 0; less than 10% = 1; 10%–25% = 2; 26%–50% = 3; 51%–75% = 4; and more than 75% = 5 (Cortes et al., 2018). Fibrosis area in Masson staining was calculated by ImageJ software (NIH, Bethesda, MD, United States).

### Metabolite extraction

A 25 mg kidney tissue sample was weighed into an EP tube. After adding 200 μL water and 480 μL extract solution (MTBE: MeOH = 5: 1), the samples were vortexed for 30 s and then homogenized at 35 Hz for 4 min and sonicated for 5 min in an ice-water bath. This cycle was repeated 3 times. The samples were then centrifuged at 3,000 rpm for 15 min at 4°C after being incubated for 1 h at −40°C. Then, 300 μL of supernatant was transferred into a new tube and dried in a vacuum concentrator at 37°C. Afterward, the dried samples were reconstituted with 200 μL of 50% methanol in dichloromethane and then centrifuged at 13,000 rpm for 15 min at 4°C. For LC/MS analysis, 75 μL of the supernatant was transferred to a new glass vial. To make a quality control (QC) sample, 20 μL of the supernatants from all of the samples were mixed together.

### LC-MS/MS analysis

A UHPLC system (1290, Agilent Technologies) with a Kinetex C18 column (2.1 × 100 mm, 1.7 μm, Phenomen) was used for the LC-MS/MS analyses. Mobile phase A contained 40% water, 60% acetonitrile, and 10 mmol/L ammonium formate. Mobile phase B contained 10% acetonitrile and 90% isopropanol. The analysis was performed using the following elution gradient, 0–12.0 min, 40%–100% B; 12.0–13.5 min, 100% B; 13.5–13.7 min, 100%–40% B; 13.7–18.0 min, 40% B. The column temperature was 55°C. The autosampler temperature was 4°C, and the injection volume was 2 μL (pos or neg).

MS/MS spectra were acquired by a QE mass spectrometer in data-dependent acquisition (DDA) mode. The ESI source conditions were as follows: sheath gas flow rate was 30 Arb, Aux gas flow rate was 10 Arb, capillary temperature was 320°C (positive) or 300°C (negative), full MS resolution was 70,000, MS/MS resolution was 17,500, collision energy was 15/30/45 in NCE mode, and spray voltage was 5 kV (positive) or −4.5 kV (negative).

### Data processing

Using ProteoWizard’s ‘msconvert’ program, raw data files were converted to mzXML files. We first applied peak detection to the MS1 data using the CentWave algorithm in XCMS to detect peaks in the MS/MS spectra. The cutoff for annotation was set at 0.3. The qualitative matching of lipids is based on three main dimensions: 1) the molecular weight of the metabolite based on the mass-to-charge ratio (m/z) of the parent ion in the primary mass spectrum; 2) the mass-to-charge ratio of the characteristic daughter ions generated after fragmentation; and 3) the response intensity of the daughter ions. These three dimensions are compared with the lipid database LipidBlast to achieve secondary identification of metabolites and to calculate substance matching scores. The software used for the characterization was XCMS, which was used for retention time correction, peak identification, peak extraction, peak integration, and peak alignment (Smith et al., 2006). Data containing peak number, sample name, and normalized peak area were imported into SIMCA16.0.2 (Sartorius Stedim Data Analytics AB, Umea, Sweden) for multivariate analysis. To minimize both noise and the high variance of the variables, the data were scaled and logarithmically transformed. Then, principal component analysis (PCA) and orthogonal projections to latent structures-discriminate analysis (OPLS-DA) were performed to visualize the distribution of the samples. Furthermore, the value of variable importance in the projection (VIP) of the first principal component in OPLS-DA was obtained. The screening criterion for differential lipids between groups was *p* < 0.05 (Student’s t test).

### Measurement of mitochondrial respiratory complex activity

Mitochondrial respiratory complex activity was tested using mitochondrial complex I, II, III, and IV kits (Jonln, Shanghai, China), following the manufacturer’s instructions. Briefly, 10 µL sample and 200 µL working solution were sequentially added to a 96-well plate, and the optical density values initially and after 2 min were read to calculate the complex activity. The wavelengths read for complexes I, II, III, and IV were 340 nm, 605 nm, 550 nm, and 550 nm, respectively.

### Western blotting

Equal amounts of kidney cortical lysate were loaded onto 10% SDS-PAGE gels, subjected to electrophoresis and membrane transfer, then blocked with 5% nonfat milk. The membranes were incubated with primary antibodies against carnitine palmitoyltransferase 1A (CPT1A), acyl-coenzyme A oxidase 1 (ACOX1), long-chain specific acyl-CoA dehydrogenase (ACADL), and glyceraldehyde-3-phosphate dehydrogenase (GAPDH) (Proteintech, Wuhan, China) at 4 °C overnight. Then, the membranes were sequentially incubated with secondary antibodies and ECL luminescent solution to visualize the protein bands. The gray values of the bands were calculated by Image Lab software version 5.1 (Bio-Rad Laboratories, Hercules, CA, United States).

### Statistical analysis

The data are presented as mean ± SEM. One-way ANOVA with Dunnett *post hoc* analysis was conducted to compare significant differences among groups using GraphPad Prism 9 (La Jolla, CA, United States). A *p* value of less than 0.05 was considered significantly different.

## Results

### HKL alleviated adenine-induced CKD in rats

**FIGURE 1:**
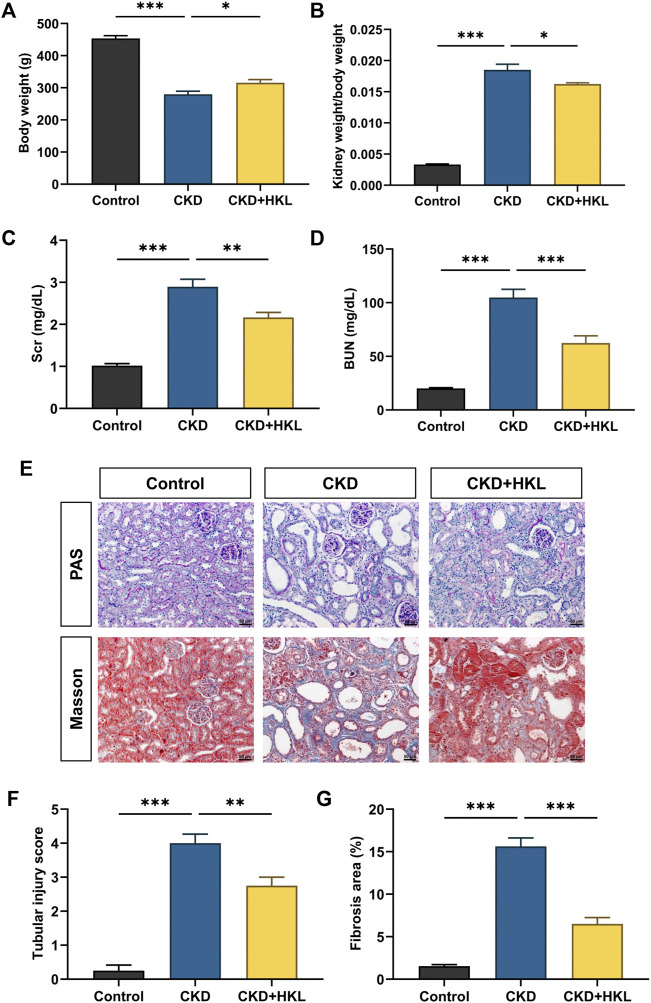
Effects of HKL on adenine-induced CKD rats. **(A)** Body weight. **(B)** Kidney weight to body weight ratio. **(C)** Serum creatinine. **(D)** Blood urea nitrogen. **(E)** PAS and Masson staining. All images are shown at identical magnification, ×200, scale bar = 50 μm. **(F)** Renal tubular injury score. **(G)** Quantification of tubulointerstitial fibrosis. Data are expressed as mean ± SEM, n = 4-6 rats per group, ^*^ *p* < 0.05, ^**^ *p* < 0.01, ^***^ *p* < 0.001.

**FIGURE 2:**
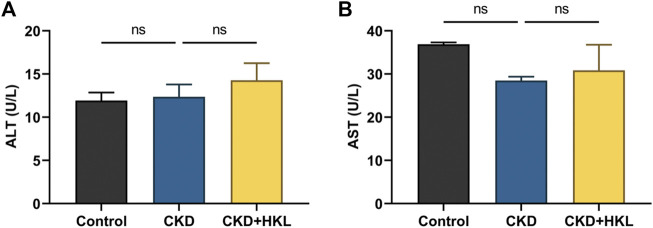
Effects of HKL on liver function indexes. **(A)** Alanine transaminase levels. **(B)** Aspartate transaminase levels. Data are expressed as mean ± SEM, n = 6 rats per group, ns = no significance.

HKL treatment attenuated body weight loss in CKD rats and reduced the kidney weight to body weight ratio (Figures 1A,B). CKD is characterized by decreased renal function and tubulointerstitial fibrosis. As shown in Figure 1, CKD rats showed elevated levels of Scr and BUN, marked renal tubule dilatation and interstitial fibrosis. HKL treatment reduced the levels of Scr (2.2 ± 0.1 mg/dl vs. 2.9 ± 0.2 mg/dl, *p* < 0.01) and BUN (62.5 ± 6.7 mg/dl vs. 104.8 ± 7.7 mg/dl, *p* < 0.001) in CKD rats (Figures 1C,D). Renal pathological staining further confirmed that HKL reduced renal tubular epithelial cell shedding and fiber deposition (Figures 1E–G). Serum ALT and AST levels were not significantly different among the three groups (Figure 2), indicating that HKL had no obvious liver toxicity. These data indicated that the administration of HKL alleviated adenine-induced CKD.

### HKL altered lipid profiles in CKD rats

**FIGURE 3:**
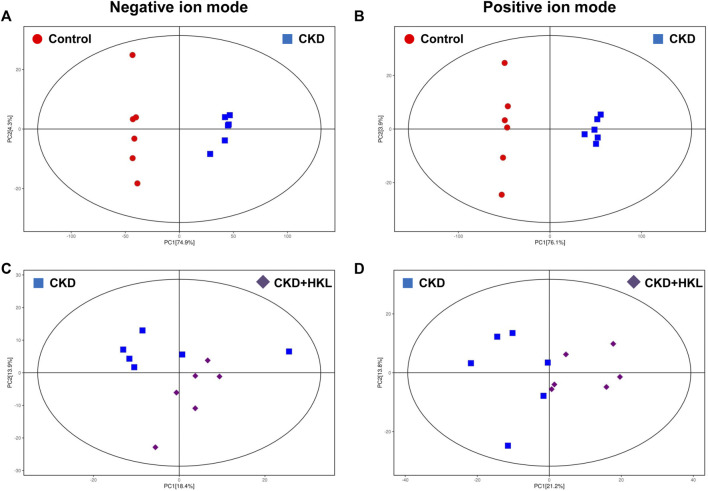
Score scatter plots of PCA models. **(A)** PCA score plot of the control and the CKD group in negative ion mode. **(B)** PCA score plot of the control and the CKD group in positive ion mode. **(C)** PCA score plot of the CKD and the CKD + HKL group in negative ion mode. **(D)** PCA score plot of the CKD and the CKD + HKL group in positive ion mode. The red dots represent the control group, the blue squares represent the CKD group, and the purple diamonds represent the CKD + HKL group.

**FIGURE 4:**
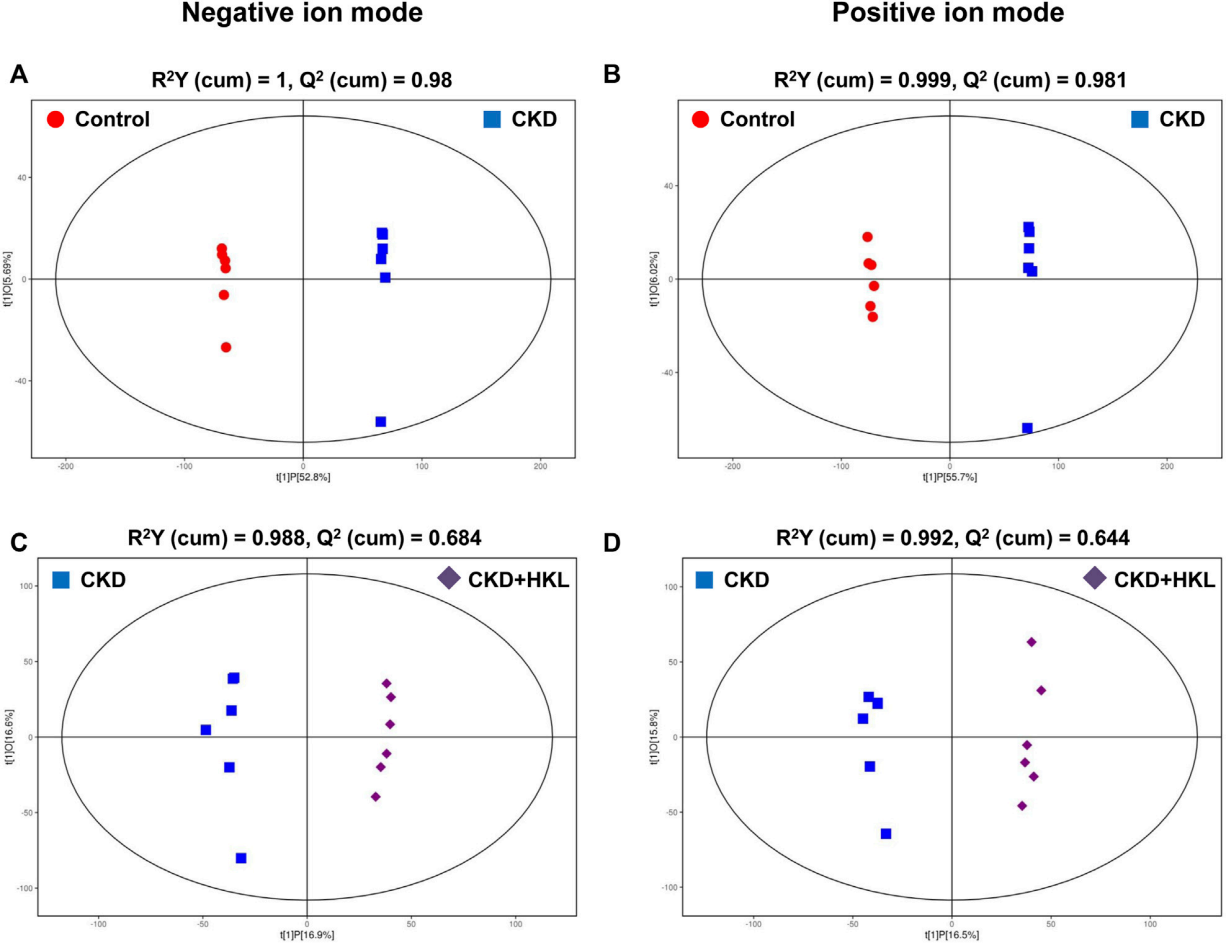
Score scatter plots of OPLS-DA model. **(A)** OPLS-DA score plot of the control and the CKD group in negative ion mode. **(B)** OPLS-DA score plot of the control and the CKD group in positive ion mode. **(C)** OPLS-DA score plot of the CKD and the CKD + HKL group in negative ion mode. **(D)** OPLS-DA score plot of the CKD and the CKD + HKL group in positive ion mode. The red dots represent the control group, the blue squares represent the CKD group, and the purple diamonds represent the CKD + HKL group.

**FIGURE 5:**
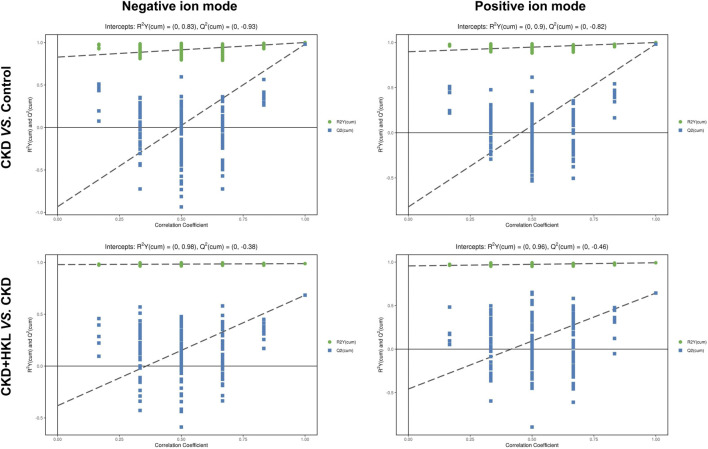
Permutation test of OPLS-DA. Permutation plots of OPLS-DA models for group CKD vs. control and group CKD + HKL vs. CKD in negative and positive ion modes.

To visually display the differences in lipid profiles between groups, PCA and OPLS-DA models were established in negative and positive ion modes. Each scatter plot represents a sample, and the distance between the scatter plots represents the difference in lipid profile between samples. In the PCA models, the scatter plots of the control group and the CKD group were significantly separated (Figures 3A,B), while the CKD group and the CKD + HKL group partially overlapped (Figures 3C,D). In the more reliable OPLS-DA models, the scatter plots were clearly divided into two clusters, whether it was the comparison between the control group and the CKD group or the comparison between the CKD group and the CKD + HKL group (Figure 4). In permutation test (n = 200) of OPLS-DA, the R^2^Y values were very close to 1, indicating that the established models conform to the real situation of the sample data (Figure 5). These results indicated that CKD rats had abnormal lipid metabolism, which could be affected by HKL treatment.

### Identification of lipids reversed by HKL treatment in CKD rats

**FIGURE 6:**
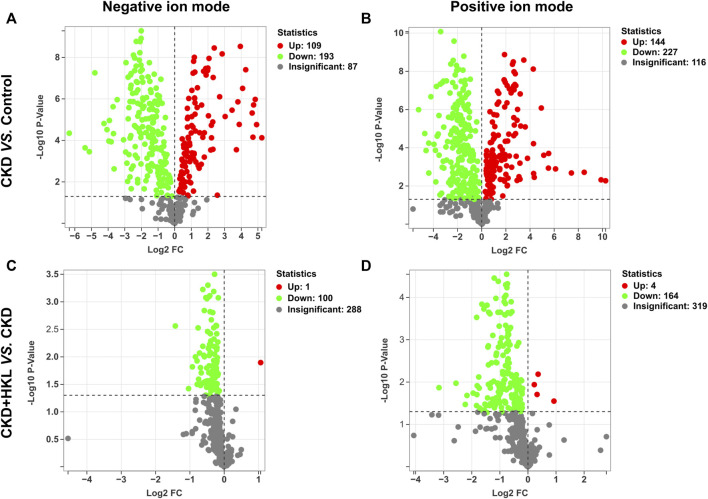
Volcano plots of lipid differences between groups. **(A)** Differential lipid profiles in CKD versus control in negative ion mode. **(B)** Differential lipid profiles in CKD versus control in positive ion mode. **(C)** Differential lipid profiles in CKD + HKL versus CKD in negative ion mode. **(D)** Differential lipid profiles in CKD + HKL versus CKD in positive ion mode. The red dots represent significant upregulation, the green dots represent significant downregulation, and the grey dots represent insignificant.

In total, 109 lipids were upregulated and 193 lipids were downregulated in the CKD group compared with the control group in negative ion mode (Figure 6A). Moreover, 144 lipids were upregulated and 227 lipids were downregulated in the CKD group in positive ion mode (Figure 6B). Only 1 lipid was upregulated and 100 lipids were downregulated in the CKD + HKL group compared with the CKD group in negative ion mode (Figure 6C). Four lipids were upregulated and 164 lipids were downregulated in the CKD + HKL group in positive ion mode (Figure 6D).

**FIGURE 7:**
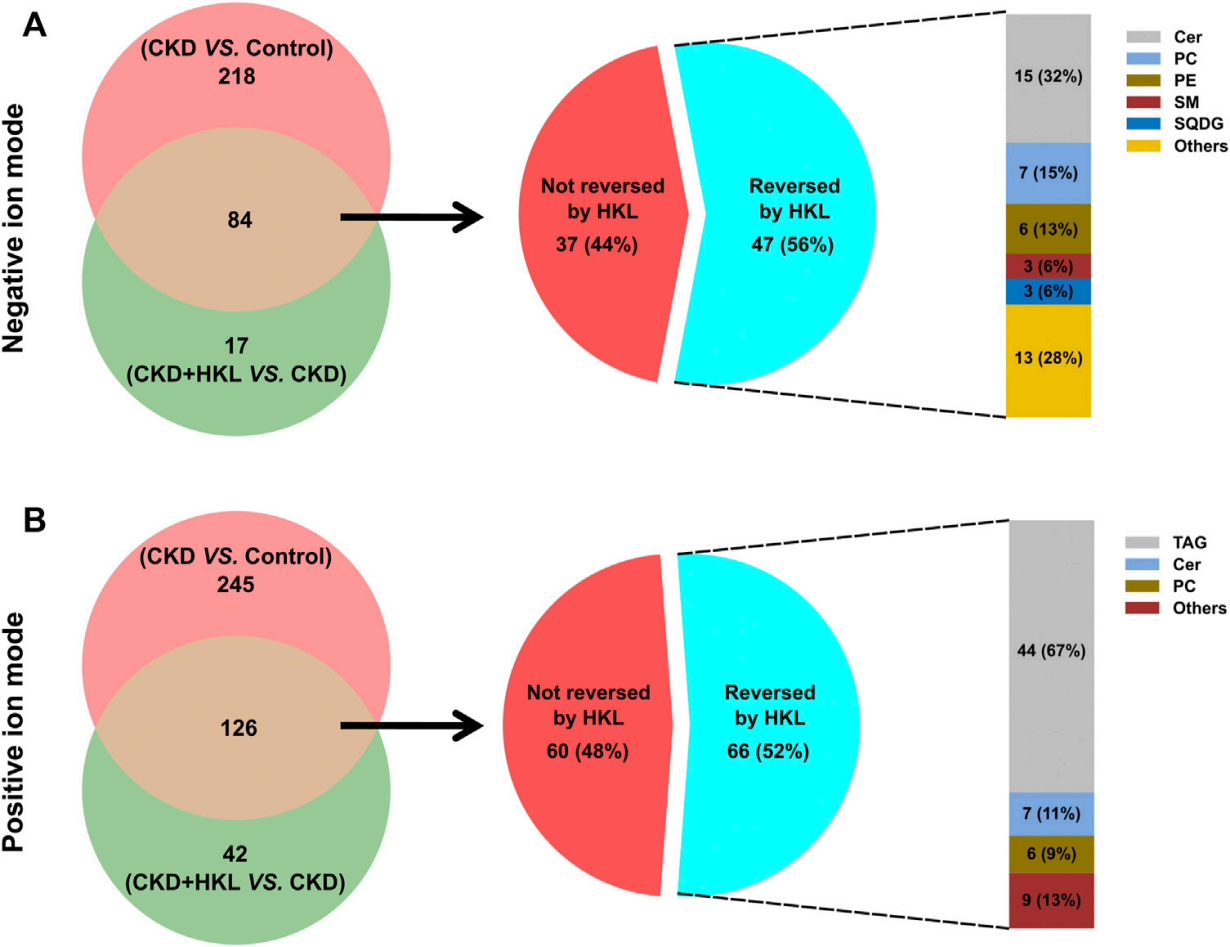
Screening of lipids that could be reversed by HKL treatment in CKD rats. **(A)** The amount and classification of lipids that could be reversed by HKL treatment in negative ion mode. **(B)** The amount and classification of lipids that could be reversed by HKL treatment in positive ion mode.

**FIGURE 8:**
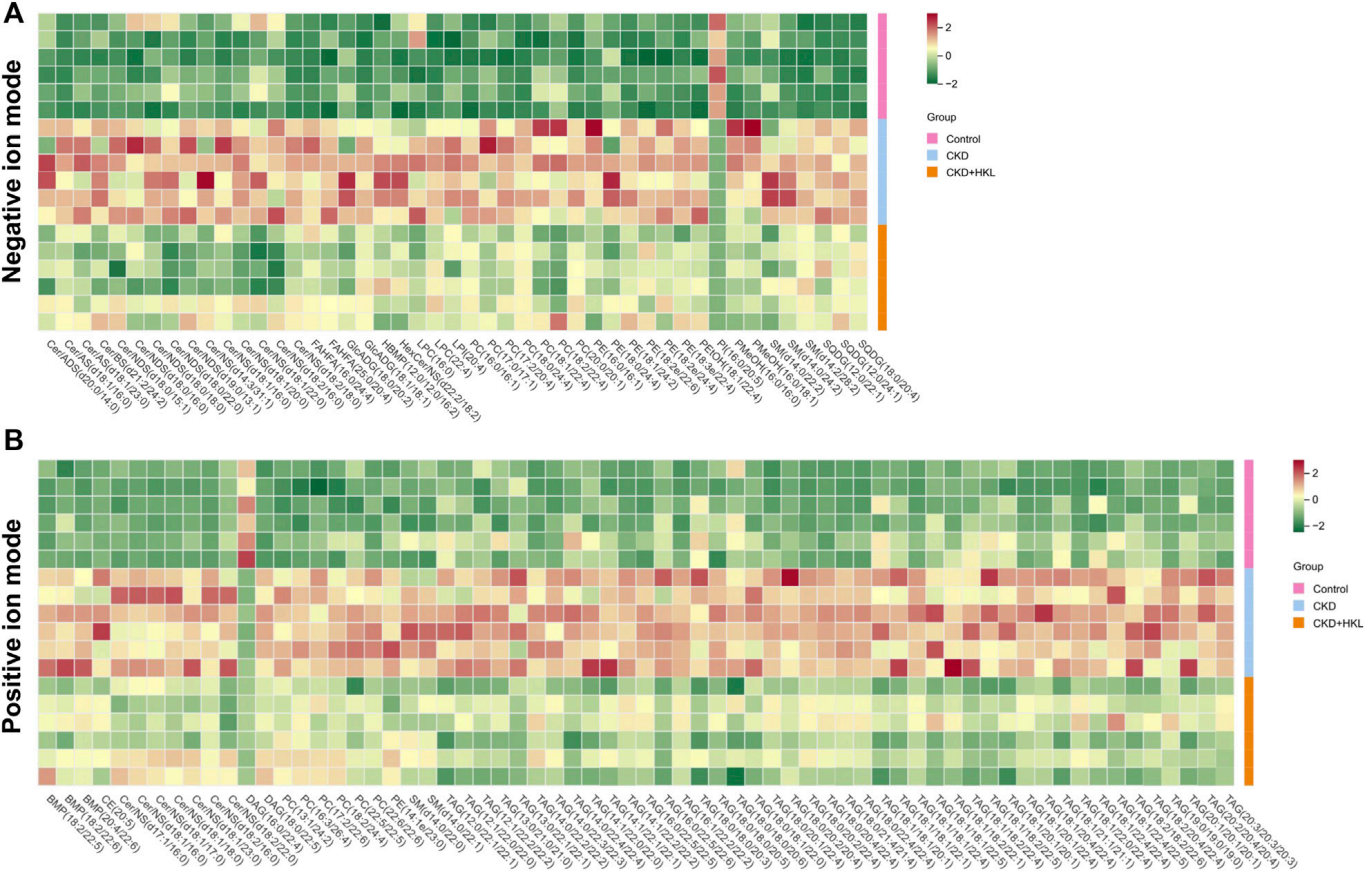
Heatmap analysis of the lipids reversed by HKL treatment in CKD rats. **(A)** Relative expression of 47 lipids in the control, CKD, and CKD + HKL group in negative ion mode. **(B)** Relative expression of 66 lipids in the control, CKD, and CKD + HKL group in positive ion mode.

The intersection of the differential lipids screened out from these two sets of comparisons resulted in 84 and 126 lipids in negative and positive ion mode, respectively (Figure 7). Further screening revealed that 47 lipids in negative ion mode could be reversed by HKL treatment; they mainly belonged to the ceramide (Cer), phosphatidylcholine (PC), and phosphatidylethanolamine (PE) classes (Figure 7A). Details of these 47 lipids are listed in Table 1. In positive ion mode, 66 lipids were reversed by HKL treatment; most of them were triacylglycerol (TAG) (Figure 7B; Table 2). Figure 8 presents the relative expression of these identified lipids in the control, CKD, and CKD + HKL groups in negative and positive ion modes. These data collectively indicated that HKL treatment could ameliorate abnormal lipid metabolism in the kidneys of CKD rats.

**TABLE 1:** The information of 47 identified lipids reversed by HKL treatment in negative ion mode.

NO.	Name	m/z	Rt (s)	CKD vs. Control			CKD + HKL vs. CKD		
				VIP	*p* value	FC	VIP	*p* value	FC
1	Cer/ADS (d20:0/14:0)	600.52	430.72	1.08	3.16E−03	1.66	1.55	3.26E−02	0.73
2	Cer/AS (d18:1/16:0)	598.50	431.33	1.36	1.70E−05	14.71	1.99	1.44E−03	0.67
3	Cer/AS (d18:1/23:0)	696.61	562.68	1.26	7.13E−05	1.75	1.85	5.97E−03	0.74
4	Cer/BS (d21:2/24:2)	700.60	555.18	1.23	1.32E−04	1.85	1.69	1.94E−02	0.73
5	Cer/NDS (d18:0/15:1)	568.49	431.35	1.29	2.29E−05	2.14	1.41	2.81E−02	0.68
6	Cer/NDS (d18:0/16:0)	584.52	473.08	1.03	7.28E−03	1.37	1.52	2.41E−02	0.83
7	Cer/NDS (d18:0/18:0)	612.56	515.41	1.09	1.84E−03	1.56	2.05	4.99E−04	0.72
8	Cer/NDS (d18:0/22:0)	668.61	574.05	1.05	3.27E−03	1.33	2.00	8.78E−04	0.71
9	Cer/NDS (d19:0/13:1)	554.48	404.73	1.22	1.70E−04	1.87	1.67	1.59E−02	0.70
10	Cer/NS (d14:3/31:1)	730.64	588.78	1.18	5.47E−03	1.84	1.56	3.57E−02	0.70
11	Cer/NS (d18:1/16:0)	536.50	455.04	1.29	1.92E−05	2.03	1.79	6.67E−03	0.74
12	Cer/NS (d18:1/20:0)	638.57	538.77	1.20	8.03E−05	1.77	2.04	5.97E−04	0.65
13	Cer/NS (d18:1/22:0)	620.60	573.98	0.91	2.13E−02	1.26	1.87	3.47E−03	0.71
14	Cer/NS (d18:2/16:0)	580.49	413.57	1.04	4.88E−03	1.28	1.74	8.27E−03	0.75
15	Cer/NS (d18:2/18:0)	608.53	463.82	1.35	1.12E−08	4.18	2.00	8.38E−04	0.69
16	FAHFA (16:0/24:4)	613.52	479.47	1.35	7.49E−05	4.63	1.56	3.25E−02	0.77
17	FAHFA (25:0/20:4)	683.59	573.98	1.26	8.92E−06	1.70	2.04	6.49E−04	0.81
18	GlcADG (18:0/20:2)	823.60	538.72	1.00	7.60E−03	1.35	1.55	2.98E−02	0.80
19	GlcADG (18:1/18:1)	795.58	489.72	1.36	1.54E−08	2.20	1.83	5.39E−03	0.88
20	HBMP (12:0/12:0/16:2)	843.58	451.92	1.25	8.44E−05	1.37	1.65	1.88E−02	0.86
21	HexCer/NS (d22:2/18:2)	776.60	452.36	1.15	9.09E−04	1.37	1.61	2.17E−02	0.86
22	LPC (16:0)	540.32	74.27	0.84	4.79E−02	1.75	1.82	8.53E−03	0.61
23	LPC (22:4)	616.36	94.56	1.32	4.30E−08	3.93	1.66	1.83E−02	0.81
24	LPI (20:4)	619.29	50.37	1.15	3.98E−04	2.06	1.50	2.68E−02	0.75
25	PC (16:0/16:1)	776.54	430.76	1.32	9.49E−07	2.29	1.81	6.50E−03	0.82
26	PC (17:0/17:1)	804.58	543.72	1.14	1.11E−03	1.56	1.72	2.05E−02	0.78
27	PC (17:2/20:4)	836.54	389.54	1.32	9.53E−07	1.80	1.94	1.57E−03	0.79
28	PC (18:0/24:4)	910.66	539.97	1.35	3.42E−08	3.64	1.61	3.08E−02	0.87
29	PC (18:1/22:4)	880.60	426.33	1.15	8.07E−04	1.35	1.91	4.76E−03	0.81
30	PC (18:2/22:4)	878.59	426.34	1.03	7.06E−03	1.24	1.67	3.43E−02	0.81
31	PC (20:0/20:1)	888.67	605.10	1.32	3.88E−07	2.05	1.53	3.12E−02	0.86
32	PE (16:0/16:1)	688.50	380.19	1.21	6.39E−04	3.43	1.84	1.52E−02	0.53
33	PE (18:0/24:4)	822.60	538.74	0.95	1.26E−02	1.32	1.48	3.95E−02	0.81
34	PE (18:1/24:2)	824.61	541.56	1.34	5.10E−08	2.32	1.81	8.95E−03	0.82
35	PE (18:2e/22:6)	772.53	427.38	1.27	7.66E−06	1.73	1.53	3.05E−02	0.86
36	PE (18:2e/24:4)	804.59	521.93	1.30	2.85E−06	1.70	1.87	2.96E−03	0.81
37	PE (18:3e/22:4)	774.54	447.48	1.35	9.88E−09	2.27	1.76	3.80E−03	0.76
38	PEtOH (18:1/22:4)	777.55	411.40	1.24	4.38E−05	1.99	1.74	1.17E−02	0.81
39	PI (16:0/20:5)	855.50	289.52	1.35	4.51E−05	0.01	1.61	1.27E−02	2.09
40	PMeOH (16:0/16:0)	661.48	375.64	1.24	7.10E−05	3.23	1.98	2.98E−03	0.59
41	PMeOH (16:0/18:1)	687.50	379.81	1.17	1.42E−03	3.03	1.73	3.16E−02	0.58
42	SM (d14:0/22:2)	773.58	406.86	0.87	3.00E−02	1.23	1.48	3.04E−02	0.82
43	SM (d14:0/24:2)	801.61	457.32	1.29	4.67E−04	2.24	1.62	2.04E−02	0.77
44	SM (d14:2/28:2)	853.53	490.15	1.31	3.52E−09	5.19	1.86	3.80E−03	0.86
45	SQDG (12:0/22:1)	819.52	260.97	1.32	2.91E−07	2.58	1.83	5.95E−03	0.76
46	SQDG (12:0/24:1)	847.55	307.08	1.36	7.07E−06	8.18	1.54	2.04E−02	0.81
47	SQDG (18:0/20:4)	869.53	275.91	1.31	9.25E−08	4.97	1.78	5.15E−03	0.76

**TABLE 2:** The information of 66 identified lipids reversed by HKL treatment in positive ion mode.

NO.	Name	m/z	Rt (s)	CKD vs. Control			CKD + HKL vs. CKD		
				VIP	*p* value	FC	VIP	*p* value	FC
1	BMP (18:2/22:5)	838.56	260.74	1.28	1.84E−06	2.02	1.54	3.91E−02	0.80
2	BMP (18:2/22:6)	836.54	245.24	1.18	2.28E−04	1.31	1.78	9.28E−03	0.86
3	BMP (20:4/22:6)	860.54	238.46	1.25	6.51E−04	2.11	1.41	4.71E−02	0.81
4	CE (20:5)	688.60	684.53	1.27	4.44E−03	9.01	1.56	3.95E−02	0.52
5	Cer/NS (d17:1/16:0)	524.50	431.63	1.24	2.21E−05	2.59	1.52	3.09E−02	0.71
6	Cer/NS (d18:1/16:0)	538.52	455.28	1.28	5.66E−04	2.32	1.59	2.56E−02	0.74
7	Cer/NS (d18:1/17:0)	552.53	478.74	1.29	1.99E−04	3.35	1.52	3.58E−02	0.75
8	Cer/NS (d18:1/18:0)	566.55	500.04	1.29	8.84E−05	2.89	1.87	6.74E−03	0.66
9	Cer/NS (d18:1/23:0)	636.63	591.40	1.26	2.01E−05	1.97	1.63	2.20E−02	0.75
10	Cer/NS (d18:2/16:0)	536.50	431.62	1.12	1.98E−04	46.63	1.89	6.02E−03	0.61
11	Cer/NS (d18:2/22:0)	620.60	545.25	1.14	3.73E−03	1.42	1.73	1.23E−02	0.73
12	DAG (16:0/22:4)	662.57	535.23	1.29	3.19E−04	0.30	1.83	6.52E−03	1.28
13	DAG (18:0/22:5)	688.62	556.35	1.29	9.30E−08	6.41	1.73	1.91E−02	0.74
14	PC (13:1/24:2)	798.59	489.58	1.27	4.51E−06	1.85	1.44	3.33E−02	0.86
15	PC (16:3/26:4)	860.61	404.99	1.24	5.22E−07	2.44	1.69	1.40E−02	0.85
16	PC (17:2/22:6)	816.56	406.10	1.12	2.87E−05	2.09	1.59	2.57E−02	0.83
17	PC (18:2/24:4)	862.62	499.96	1.19	5.11E−05	1.76	1.71	1.40E−02	0.85
18	PC (22:5/22:5)	882.60	389.37	1.16	1.44E−04	1.33	1.99	1.17E−03	0.80
19	PC (22:6/22:6)	878.56	370.12	1.30	4.05E−07	2.66	2.22	1.27E−04	0.66
20	PE (14:1e/23:0)	746.60	497.53	1.18	2.84E−04	1.34	1.45	4.04E−02	0.87
21	SM (d14:0/22:0)	733.61	452.27	1.10	1.23E−03	1.31	1.65	1.70E−02	0.86
22	SM (d14:0/22:1)	731.60	452.24	1.14	5.02E−04	1.30	1.68	1.42E−02	0.88
23	TAG (12:0/22:1/22:1)	937.82	762.72	0.99	4.82E−03	1.41	2.02	1.29E−03	0.62
24	TAG (12:1/22:0/22:0)	939.83	776.22	1.14	7.20E−04	1.86	1.87	4.67E−03	0.62
25	TAG (12:1/22:2/22:2)	931.76	708.92	1.09	1.67E−04	1.81	2.21	5.05E−05	0.58
26	TAG (13:0/21:0/21:0)	927.82	727.21	1.09	3.55E−04	2.04	2.14	1.27E−04	0.56
27	TAG (13:0/22:1/22:1)	951.82	710.51	1.03	4.54E−03	2.90	2.03	7.61E−03	0.30
28	TAG (14:0/22:2/22:2)	961.82	755.66	1.22	3.62E−06	7.61	1.64	2.36E−02	0.67
29	TAG (14:0/22:3/22:3)	957.77	709.98	1.10	9.84E−07	7.83	1.96	9.04E−04	0.52
30	TAG (14:0/22:4/22:4)	953.75	705.50	1.00	4.99E−03	1.52	2.22	9.96E−05	0.56
31	TAG (14:1/22:0/22:0)	967.86	789.33	1.04	3.08E−03	1.60	1.95	2.82E−03	0.61
32	TAG (14:1/22:1/22:1)	963.83	763.25	1.14	4.38E−04	1.75	1.77	7.34E−03	0.67
33	TAG (14:1/22:2/22:2)	959.80	740.56	1.31	4.29E−09	5.85	1.97	7.04E−04	0.61
34	TAG (16:0/22:5/22:5)	977.83	721.27	1.16	1.52E−07	9.12	2.08	2.61E−04	0.57
35	TAG (16:0/22:5/22:6)	970.78	679.83	1.05	1.62E−03	2.03	2.17	1.88E−04	0.34
36	TAG (16:1/22:2/22:2)	987.83	757.63	1.24	1.06E−08	6.39	2.09	2.40E−04	0.65
37	TAG (18:0/18:0/20:3)	935.80	748.24	0.78	4.48E−02	1.28	2.00	1.76E−03	0.61
38	TAG (18:0/18:0/20:5)	931.77	726.06	1.18	7.75E−06	2.12	2.18	4.77E−05	0.60
39	TAG (18:0/18:0/20:6)	929.76	710.81	0.86	1.97E−02	1.23	1.79	1.06E−02	0.65
40	TAG (18:0/18:1/22:0)	962.91	789.56	1.19	1.30E−04	1.87	2.00	1.09E−03	0.62
41	TAG (18:0/20:1/22:4)	982.88	756.10	1.20	7.35E−07	3.74	1.98	7.58E−04	0.62
42	TAG (18:0/20:2/20:4)	952.83	725.80	1.24	2.46E−03	4.01	2.07	1.35E−02	0.49
43	TAG (18:0/20:2/22:4)	980.86	741.76	1.29	3.21E−09	6.13	2.03	5.59E−04	0.63
44	TAG (18:0/20:4/22:4)	976.83	721.75	1.26	2.58E−09	11.21	2.08	2.57E−04	0.56
45	TAG (18:0/21:4/21:4)	981.79	720.91	1.26	5.61E−08	7.10	2.10	1.40E−04	0.57
46	TAG (18:0/22:4/22:4)	1004.86	720.66	1.23	4.90E−08	6.70	2.16	8.93E−05	0.58
47	TAG (18:1/18:1/20:1)	930.85	748.24	0.85	2.23E−02	1.40	2.05	8.56E−04	0.60
48	TAG (18:1/18:1/22:1)	958.88	763.32	1.12	1.05E−03	1.89	2.05	1.41E−03	0.54
49	TAG (18:1/18:1/22:4)	957.79	725.60	1.29	5.56E−08	4.30	2.24	5.92E−05	0.60
50	TAG (18:1/18:1/22:5)	955.77	708.65	1.09	3.32E−03	2.05	1.85	1.12E−02	0.51
51	TAG (18:1/18:2/22:1)	956.86	741.10	1.17	1.43E−03	2.81	1.82	1.41E−02	0.56
52	TAG (18:1/18:2/22:4)	950.73	679.21	1.03	3.07E−03	1.95	2.18	2.45E−04	0.39
53	TAG (18:1/18:2/22:5)	948.80	705.75	0.97	9.48E−03	1.71	2.12	1.50E−03	0.48
54	TAG (18:1/20:1/20:1)	958.87	747.73	1.00	2.16E−03	1.40	2.06	5.17E−04	0.65
55	TAG (18:1/20:1/22:4)	980.86	725.67	1.28	3.78E−08	3.86	2.24	2.85E−05	0.59
56	TAG (18:1/20:4/22:4)	974.82	705.56	1.21	4.44E−05	4.44	2.19	5.67E−03	0.54
57	TAG (18:1/21:1/21:1)	986.91	763.75	1.06	9.94E−04	1.53	2.13	2.30E−04	0.61
58	TAG (18:1/22:0/22:4)	1010.91	773.84	1.29	5.95E−08	6.20	1.78	7.08E−03	0.67
59	TAG (18:1/22:4/22:4)	1002.93	749.31	0.93	4.40E−03	1.87	2.11	5.87E−04	0.61
60	TAG (18:1/22:4/22:5)	1000.83	700.72	1.11	4.51E−04	3.12	1.80	1.51E−02	0.53
61	TAG (18:2/18:2/22:6)	949.72	678.94	1.08	8.96E−04	2.22	2.05	7.93E−04	0.42
62	TAG (18:2/20:4/22:4)	972.80	700.01	1.18	2.67E−04	3.12	1.99	2.36E−03	0.46
63	TAG (19:0/19:0/19:0)	955.85	740.72	1.24	6.39E−04	8.97	1.74	1.52E−02	0.59
64	TAG (20:1/20:1/20:1)	991.86	777.43	1.06	2.20E−03	1.60	1.98	1.89E−03	0.59
65	TAG (20:2/20:4/20:4)	977.75	698.61	1.24	2.69E−04	4.89	2.22	2.25E−04	0.38
66	TAG (20:3/20:3/20:3)	979.77	704.94	1.26	1.35E−07	5.40	2.08	2.25E−04	0.51

Abbreviations: BMP, bismonoacylglycerophosphate; CE, cholesteryl ester; Cer/NS, ceramide non-hydroxyfatty acid-sphingosine; CKD, chronic kidney disease; DAG, diacylglycerol; FC, fold change; HKL, honokiol; PC, phosphatidylcholine; PE, phosphatidylethanolamine; SM, sphingomyelin; TAG, triacylglycerol; VIP, variable importance in the projection.

### HKL improved fatty acid oxidation in CKD rats

**FIGURE 9:**
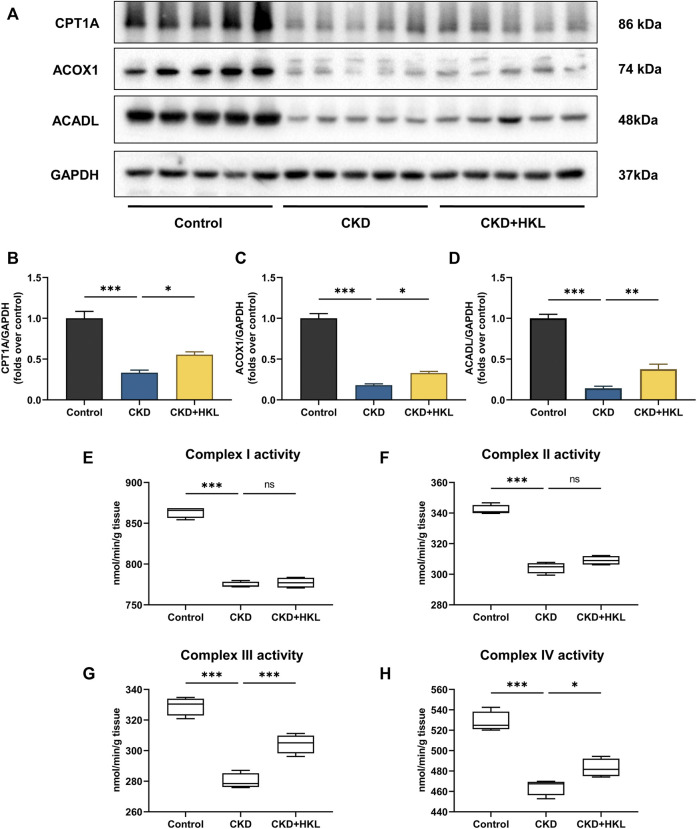
Effects of HKL on fatty acid oxidation in the kidney of CKD rats. **(A)** Representative Western blot bands of CPT1A, ACOX1, and ACADL expression in the kidneys of rats. **(B**–**D)** Densitometric analysis of CPT1A, ACOX1, and ACADL normalized to GAPDH content. Data are expressed as mean ± SEM, n = 5. **(E**–**H)** The activity of mitochondrial respiratory complex I, II, III, and IV in the kidney of rats. Data are expressed as min to max, n = 4. ^*^ *p* < 0.05, ^**^ *p* < 0.01, ^***^ *p* < 0.001, ns = no significance.

Impaired renal fatty acid oxidation (FAO), a hallmark of CKD, leads to lipid accumulation and induces lipid nephrotoxicity. As shown in Figure 9, the expression of key enzymes involved in FAO and mitochondrial respiratory complex activity were both significantly decreased in the kidneys of CKD rats (*p* < 0.001). HKL treatment upregulated the expression of key FAO enzymes and increased the activity of complexes III and IV in CKD rats. These results suggested that HKL could partially restore FAO in the kidneys of CKD rats.

## Discussion

In the present study, HKL was found to reduce the levels of Scr and BUN and improve renal pathological injury in an adenine-induced CKD rat model. CKD rats showed abnormal lipid abundance in the kidney, which could be partially restored by HKL treatment. These lipids mainly belonged to the PC, Cer, PE, and TAG classes.

HKL has a variety of pharmacological effects (Rauf et al., 2021). HKL has primarily been studied for its effects on acute kidney injury models through its anti-inflammatory and anti-oxidative stress properties (Li et al., 2014; Yu et al., 2016; Xia et al., 2019; Zhang and Xiang, 2019; Park et al., 2020). Liu et al. treated cisplatin-induced chronic kidney injury mice with 5 mg/kg nanosized liposome-encapsulated HKL for 6 weeks. The results showed that HKL could mitigate chronic kidney injury by preserving mitochondrial antioxidant capacity and reducing apoptosis (Liu et al., 2019). The present study used an adenine-induced CKD rat model that mimics the majority of the structural and functional changes seen in human CKD. (Diwan et al., 2018). Similarly, HKL was proven to protect kidney structure and function (Figure 1). Together with previous studies, this study expands the application of HKL in kidney disease and provides an option for the treatment of CKD.

By using lipidomics, we identified 102 lipids (43 lipids in negative ion mode, 59 lipids in positive ion mode) that could be reversed by HKL treatment in CKD rats. Most of these lipids belonged to the PC, Cer, PE, and TAG classes (Figure 7). PCs and PEs are the most abundant phospholipids in all mammalian cell membranes (Van der Veen et al., 2017). PEs can be converted to PCs *via* the catalysis of PE N-methyltransferase (Vance and Tasseva, 2013). Several studies have identified associations between PCs/PEs and CKD. Tofte et al. reported that PCs were associated with renal impairment and all-cause mortality in type 1 diabetes (Tofte et al., 2019). In the Chronic Renal Insufficiency Cohort, PEs were higher in progressive CKD patients than in nonprogressors (Afshinnia et al., 2016). In the Clinical Phenotyping and Resource Biobank Core cohort, a higher abundance of PCs and PEs was independently associated with a higher risk of stroke in CKD (Afshinnia et al., 2020). Ceramides are an important family of bioactive lipids belonging to the sphingolipid family and comprise sphingosine and an amide-linked fatty acid (Aburasayn et al., 2016). Ceramide levels were increased in children with CKD (Mitsnefes et al., 2014). Increased levels of plasma ceramides were associated with CKD independent of other established risk factors (Mantovani et al., 2021). Mechanistically, ceramide-accumulation-induced apoptosis and redox signaling contribute to the development of renal dysfunction (Li and Zhang, 2013; Ueda, 2015). CKD is associated with increased levels of circulating TAGs, which are thought to increase the risk of atherosclerotic cardiovascular disease in the CKD population (Lamprea-Montealegre et al., 2020). Notably, the TAGs we identified had longer fatty acid acyl chains and multiple double bonds (Table 2). According to a previous study, TAGs with longer acyl chains and more double bonds were more abundant as the CKD stages progressed (Afshinnia et al., 2018).

Growing evidence has indicated that lipid accumulation and its consequent lipotoxicity are definite contributors to CKD (Chen et al., 2022). FAO is a major source of ATP generation of proximal tubule, and defective FAO has a key role in kidney fibrosis development (Kang et al., 2015). Our data found that HKL treatment alleviated lipid accumulation in the kidney of CKD rat (Figures 6, 8). Moreover, HKL upregulated the expression of FAO key enzymes (Figure 9), suggesting that HKL reduced renal lipid deposition by increasing FAO. In methionine-choline deficient diet-induced hepatic steatosis mice model, HKL also attenuated hepatic lipid deposition by promoting FAO (Zhai et al., 2020). Previous studies have reported that HKL can regulate lipid metabolism through other mechanisms. Liu et al. found that HKL could ameliorate lipotoxicity in hepatocytes by activating SIRT3-AMPK mediated lipophagy (Liu et al., 2021). Additional study suggested that HKL could block fatty acid synthesis by inhibiting sterol regulatory element-binding protein-1c (SREBP-1c) maturation and the induction of lipogenic proteins, stearoyl-CoA desaturase-1 (SCD-1) and fatty acid synthase (FASN) (Seo et al., 2015). Whether HKL can regulate lipid metabolism through other mechanisms in adenine-induced CKD needs further investigation.

## Conclusion

In conclusion, HKL could protect against adenine-induced CKD, possibly through the regulation of lipid metabolism.

## Data Availability

The original contributions presented in the study are included in the article/supplementary material, further inquiries can be directed to the corresponding authors.

## Abbreviations

Abbreviations: Cer/ADS, ceramide alpha-hydroxy fatty acid-dihydrosphingosine; Cer/AS, ceramide alpha-hydroxy fatty acid-sphingosine; Cer/BS, ceramide beta-hydroxy fatty acid-sphingosine; Cer/NDS, ceramide non-hydroxyfatty acid-dihydrosphingosine; Cer/NS, ceramide non-hydroxyfatty acid-sphingosine; CKD, chronic kidney disease; FAHFA, fatty acid ester of hydroxyl fatty acid; FC, fold change; GlcADG, glucuronosyldiacylglycerol; HBMP, hemibismonoacylglycerophosphate; HexCer/NS, hexosylceramide non-hydroxyfatty acid-sphingosine; HKL, honokiol; LPC, lysophophatidylcholine; LPI, lysophosphatidylinositol; PC, phosphatidylcholine; PE, phosphatidylethanolamine; PEtOH, phosphatidylethanol; PI, phosphatidylinositol; PMeOH, phosphatidylmethanol; SM, sphingomyelin; SQDG, sulfoquinovosyl diacylglycerol; VIP, variable importance in the projection.
